# Clinical characteristics, surgical management, and prognostic factors for supratentorial hemangioblastoma: A retrospective study

**DOI:** 10.3389/fsurg.2022.1092140

**Published:** 2023-01-24

**Authors:** Long Chen, Zujian Xiong, Yian Zhou, Yanwen Li, Yuanyang Xie, Yi Xiong, Siyi Wanggou, Xuejun Li

**Affiliations:** ^1^ Department of Neurosurgery, Xiangya Hospital, Central South University, Changsha, China; ^2^ Xiangya School of Medicine, Central South University, Changsha, China; ^3^ Hunan International Scientific and Technological Cooperation Base of Brain Tumor Research, Xiangya Hospital, Central South University, Changsha, China; ^4^ School of Mathematics and Statistics, Central South University, Changsha, China

**Keywords:** solid hemangioblastoma, supratentorial hemangioblastoma, von Hippel–Lindau disease, neuro-oncology, cystic hemangioblastoma

## Abstract

**Background:**

Supratentorial hemangioblastoma is an extremely rare neoplasm. The aim of this study is to delineate the clinical features among cystic and solid supratentorial hemangioblastoma patients and evaluate the risk factors for progression-free survival (PFS).

**Methods:**

We conducted a literature search in PubMed for histopathologically identified supratentorial hemangioblastoma between 1947 and 2021 and extracted and collected the clinical features of patients treated at our own institute. The rate of PFS was determined using Kaplan–Meier analysis. Differences in categorical factors, such as the location of tumor and diagnosis of von Hippel–Lindau disease, were analyzed using the Pearson *χ*^2^ test. A Cox regression analysis was performed to evaluate the association between various variates and survival outcomes.

**Results:**

A total of 237 cases of supratentorial hemangioblastoma were identified from 169 studies. A survival analysis found that patients with cystic tumors had a significantly better prognosis than those with solid tumors (log-rank, *p* = 0.0122). Cox regression analysis suggested that cystic hemangioblastoma (hazard ratio (HR): 0.186, 95% CI: 0.043–0.803, *p* < 0.05) and gross total resection (GTR) (HR: 0.126, 95% CI: 0.049–0.323, *p* < 0.001) were significant predictors of longer survival (PFS) for supratentorial hemangioblastoma. Following an analysis of 13 supratentorial hemangioblastoma cases from our institute, we validated that cystic tumor had improved prognosis than solid tumor (log-rank, *p* = 0.0096) and GTR was superior to subtotal resection (log-rank, *p* = 0.0029).

**Conclusions:**

Cystic hemangioblastoma vs. solid hemangioblastoma may be two tumoral statuses with different clinical features, and a specific treatment strategy should be considered.

## 1. Introduction

Hemangioblastoma, a rare tumor of the central nervous system (CNS), was first reported by von Hippel–Lindau in 1895 (1). It is a benign, vascular tumor that accounts for 1%–3% of intracranial space-occupying lesions (1, 2), and recent studies have demonstrated that hemangioblastoma arises from arrested mesoderm-derived hemangioblasts during embryonic development (3–5). Hemangioblastoma occurs frequently in the cerebellum, brainstem, and spinal cord, but rarely does supratentorial hemangioblastoma occur either sporadically or in conjunction with von Hippel–Lindau (VHL) disease, which accounts for approximately one-third of cases (6–8).

VHL disease is an autosomal-dominant inherited disorder, mainly caused by germline mutations of the VHL gene located on the short arm of chromosome 3. The prevalence of VHL is 1/(91,000–36,000) (9) and penetrance can reach 87%–97% (10, 11). A VHL patient often presents with a multiorgan tumor syndrome, including CNS hemangioblastoma, retinal hemangioblastoma, renal carcinoma or renal cyst, pancreatic neuroendocrine tumor or cysts, and adrenal pheochromocytoma (12, 13). The development and progression of CNS-associated hemangioblastoma is the most common cause of morbidity and mortality in VHL disease, among which supratentorial hemangioblastoma accounts for only 1%–6% of VHL-related hemangioblastoma (7, 14). However, studies focusing on supratentorial hemangioblastoma have been rarely reported, which makes it difficult to provide valuable information on clinical diagnosis and treatment.

This study was performed mainly to summarize the clinical characteristics of sporadic and VHL-related supratentorial hemangioblastoma and explore the differences in clinical characteristics between cystic and solid tumors in supratentorial hemangioblastoma in order to enrich the current evidence of biological and clinical features and further optimize the management of intracranial hemangioblastoma, especially supratentorial hemangioblastoma.

## 2. Materials and methods

### 2.1. Search methodology

The first author (LC) conducted a literature search in the US National Library of Medicine (PubMed) in 2021 to identify all reported cases of supratentorial hemangioblastoma (between October 1947 and March 2021). The following specific search terms were used: “hemangioblastoma,” “hemangioblastomas,” “haemangioblastoma,” “supratentorial hemangioblastoma,” “angioblastoma,” and “angioreticuloma.” A total of 2,964 articles were identified, of which 169 contained patient data regarding supratentorial hemangioblastomas.

### 2.2. Selection criteria

Publications were eligible if they met the following criteria: acquisition of full-text articles; studies or case reports containing individual patient clinical data or purely supratentorial hemangioblastoma–aggregated datasets of either histopathologically confirmed or presented in the context of a confirmed diagnosis of VHL disease. The standard Preferred Reporting Items for Systematic Reviews and Meta-Analyses of Individual Participant Data (PRISMA-IPD) methodology was used in this study.

Diagnosis of VHL was confirmed in study patients using genetic testing and/or diagnostic clinical criteria (14, 15): a family history of VHL disease with CNS hemangioblastoma, pheochromocytoma, or renal clear cell carcinoma; two or more CNS hemangioblastomas or one CNS hemangioblastoma with a visceral tumor, including pancreatic cysts, neuroendocrine tumors, and endolymphatic sac tumors, but not renal and epididymal cysts.

### 2.3. Data extraction

#### 2.3.1. Clinical evaluation

For each case, detailed clinical information [sex, age, symptom, VHL, retinal hemangioblastoma, visceral lesion, preoperation Karnofsky Performance Scale (KPS), postoperation KPS, progression-free survival (PFS), etc.] was extracted from the original article. The KPS score was mainly obtained directly through the content of the article. The postoperative KPS score is an assessment of the status of surgical patients 1 week after surgery. If it is not mentioned in the article, we will score it according to the preoperative and postoperative conditions by using the KPS. All data were entered into a dedicated form and validated by two reviewers (ZX and YX).

#### 2.3.2. Imaging evaluation

The specific imaging data of each patient were extracted, such as CT scan images, fluid attenuated inversion recovery (FLAIR), and T1- and T2-weighted MRI. The cystic hemangioblastoma in this study included peritumoral and intratumoral cysts. Peritumoral cysts were judged by using T2-weighted MRI, and intratumoral cysts were determined by using T1-weighted postcontrast MRI, which manifested as hypo-intensity within enhancing tumor. In supratentorial multiple tumors, the tumor that was the largest in terms of volume or that caused symptoms was used to define whether the patient had a cystic or solid tumor. The definition of deep and superficial anatomical locations is as follows: superficial refers to the frontal, temporal, parietal, and occipital lobes and deep denotes the sellar region, basal ganglia, ventricles, and hippocampal regions. Peritumoral edema was obtained as described from the content of the study or judged by using CT and T2 FLAIR images. Information about tumor volume was obtained from the description in the article, which refers to the total volume of cystic and solid components. If tumor volume was not mentioned in the paper, NA (not available) was entered instead. In accordance with the cases reported in our institute, tumor size evaluation was performed as previously described (16) and the extent of resection was determined, which was recorded as gross total resection (GTR) or subtotal resection (STR) on postoperative contrast MRI scans before discharge. Detailed anatomical location of the tumor and cyst was obtained from the article content; if not, we judged the specific location by image data. All information was entered into the form and reviewed by two people (SW and YX). All disagreements were resolved through consultation with a third reviewer when needed (XL).

### 2.4. Statistical analysis

For categorical data, Pearson's *χ*^2^ test, Fisher's exact test, Wilcoxon rank-sum test, or Fisher's exact test were used to analyze the differences among preoperation variates. Spearman rank correlation analysis was used in different categorical data. The data were analyzed statistically using descriptive analysis by Cramer's *V* to determine the correlation degree between the groups. The Kaplan–Meier (K–M) survival analysis method was employed to generate time–to-progression curves and these were tested by using log-rank. Cox regression analysis was performed to evaluate the association between various variates and survival outcomes. A *p*-value of <0.05 was considered statistically significant. All statistical analyses and graphics were prepared using Statistical Package for the Social Sciences (IBM SPSS Statistics 23.0) and Graphpad Prism 8.0 software.

## 3. Results

### 3.1. Clinical characteristics of supratentorial hemangioblastoma

**Figure 1:**
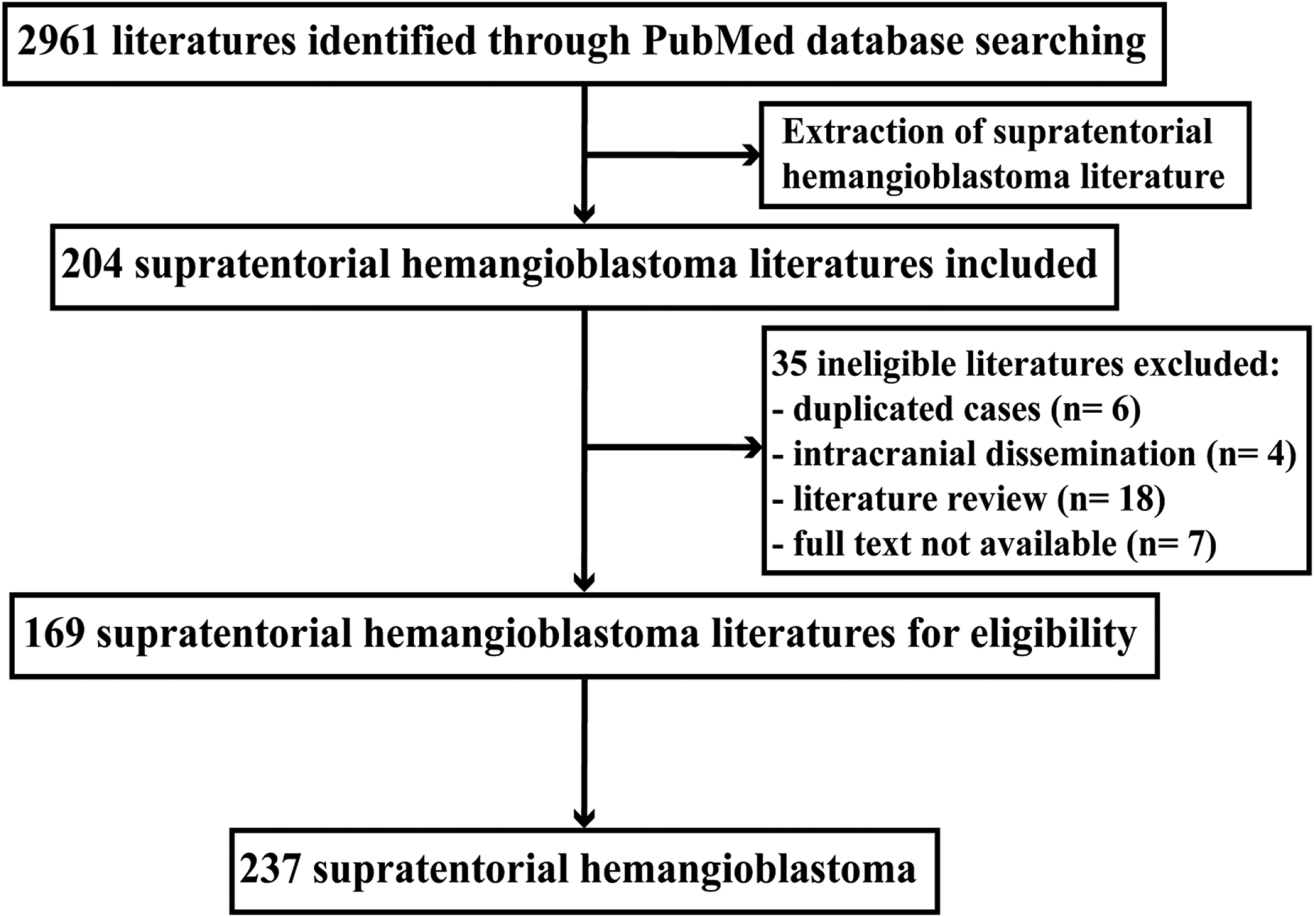
Study schema: patient selection flow chart.

Following the selection criteria (Figure 1), a total of 169 articles were retrieved, from which 237 supratentorial hemangioblastoma patients were identified, with a less female predominance (48.5%), and the mean age of onset was 38.51 ± 19.11 years (range 0.06–85.00 years) (Supplementary Figure S1A). The most common symptoms included vision deficit (29.1%), followed by headache/dizziness (24.5%), and increased intracranial pressure (ICP) (12.7%). A total of 45.1% of patients were genetically or clinically diagnosed with VHL disease (Table 1).

**Table 1:** Clinical characteristics of the study population.

Parameters	Patients (*n* = 237)
	No. (%) (95% CI)
**Demographics**	
Age at diagnosis (year), mean (95% CI)	38.5 (35.9–41.1)
**Sex**	
Male	102 (43.0) (36.6–49.6)
Female	115 (48.5) (42.0–55.1)
NA	20 (8.5)
**Clinical features**	
Presenting symptoms, No. (%)	
Headache/dizziness	58 (24.5) (19.1–30.5)
Motor/gait symptoms	19 (8.0) (4.9–12.2)
Vision deficit	69 (29.1) (23.4–35.3)
Cognitive behavioral deficit	14 (5.9) (3.3–9.7)
Seizures	24 (10.1) (6.6–14.7)
Endocrine-associated symptoms	8 (3.4) (1.5–6.5)
Asymptom	27 (11.4) (7.6–16.1)
NA	18 (7.6)
**VHL syndrome, No. (%)**	
Yes	107 (45.1) (38.7–51.7)
No	115 (48.5) (42.0–55.1)
NA	15 (6.3)
**Solitary tumor in the supratentorial area, No. (%)**	
Solitary	222 (93.7) (89.8–96.4)
Multiple	15 (6.3) (3.6–10.2)
**Infratentorial hemangioblastoma, No. (%)**	
Yes	63 (26.6) (21.1–32.7)
No	125 (52.7) (46.2–59.2)
NA	49 (20.7)
**Retinal hemangioblastoma, No. (%)**	
Yes	40 (16.9) (12.3–22.3)
No	144 (60.8) (54.2–67.0)
NA	53 (22.4)
**Visceral lesions, No. (%)^a^**	
Yes	67 (28.3) (22.4–34.5)
No	109 (46.0) (39.5–52.6)
NA	61 (25.7)
**Polycythemia, No. (%)**	
Yes	7 (3.0) (1.2–6.0)
No	86 (36.3) (30.2–42.8)
NA	144 (60.8)
**Preoperation KPS score estimation, No. (%)**	
≥70	138 (58.2) (51.7–64.6)
<70	30 (12.7) (8.7–17.6)
NA	69 (29.1)
**Postoperation KPS score estimation, No. (%) (*n* = 194)**	
≥70	119/194 (61.3) (54.1–68.2)
<70	10/194 (5.2) (2.5–9.3)
NA	65/194 (33.5)
**Increased ICP, No. (%)**	
Yes	30 (12.7) (8.7–17.6)
No	190 (80.2) (74.5–85.0)
NA	17 (7.2)
**Other diseases, No. (%)^a^**	
None reported	214/237 (90.2)
Reported	23/237 (9.8)
**Imaging features**	
**Tumor location**	
Deep	103/237 (43.5) (37.1–50.0)
Superficial	134/237 (56.5) (50.0–62.9)
**Tumor component**	
Cystic	64 (27.0) (21.5–33.1)
Solid	147 (62.0) (55.5–68.2)
NA	26 (11.0)
**Edema (CT scan and/or MRI)**	
Yes	57 (24.1) (18.8–30.0)
No	66 (27.8) (22.2–34.0)
NA	114 (48.1)
**Flow void effect (MRI)**	
Yes	32 (13.5) (9.4–18.5)
No	50 (21.1) (16.1–26.8)
NA	155 (65.4)
**Hemorrhage (CT scan or MRI)**	
Yes	15 (6.3) (3.6–10.2)
No	154 (65.0) (58.5–71.0)
NA	68 (28.7)
**Management features**	
Treatment modality, No. (%)	
Surgery	187 (78.9) (73.2–83.9)
GTR	135 (57.0) (50.4–63.4)
STR	27 (11.4) (7.6–16.1)
NA	25 (10.5) (6.9–15.2)
RT	12 (5.1) (2.6–8.7)
Combined treatment (6 RT + STR, 1 RT + GTR)	7 (3.0) (1.2–6.0)
Conservative	31 (13.1) (9.1–18.0)

RT, radiotherapy; CI, confidence interval; VHL, von Hippel–Lindau; KPS, Karnofsky Performance Scale; ICP, intracranial pressure; STR, subtotal resection.

^a^ See Supplementary Table S1 for visceral lesion and other diseases.

Of the 237 patients, 222 (93.7%) had supratentorial solitary hemangioblastoma, the remaining 15 (6.3%) had supratentorial hemangioblastomas with multiple foci, and the total number of patients with tumors was 260. Of these patients, 147 (62.0%) had solid tumors, and 64 (27.0%) were classified as having cystic tumors, which accounted for approximately one-third of the patients (Table 1). Among patients with VHL, the presence of extracranial tumors was also reported, of which renal cell carcinoma is the most common, followed by adrenal pheochromocytoma (Supplementary Table S1). In supratentorial hemangioblastoma, the cerebrum (36.3%) is the most common location, followed by the sellar region (24.5%) (Supplementary Table S2). Notably, dual attachment (10.5%) and calcification (2.5%) can also be seen in CT imaging and MRI for supratentorial hemangioblastoma (Supplementary Table 2). The total follow-up of these cases ranged from 0 to 276 months (Supplementary Table S3).

Of these cases with available treatment modality, 187 (78.9%) patients underwent surgery alone, and 12 (5.1%) and 31 (13.1%) received radiotherapy and conservative treatment, respectively (Table 1). Among the 187 surgically treated patients, 27 (11.4%) underwent STR.

### 3.2. Subgroup analysis stratified by age or solid/cystic tumor

An analysis of the distribution of supratentorial hemangioblastoma stratified by age subgroup showed that, at the age subgroup range of 0–20 years, the optic nerve was the least frequently involved, whereas the ventricular nerve was rare in the age subgroup of more than 20 years (Supplementary Table S4). A further exploration of the tumor distribution in different locations suggested that the frontal (32.6%) and temporal (31.4%) lobes were the most frequently involved in the cerebrum, least in the occipital lobe (7.0%), whereas the sellar region was mainly suprasellar (44.8%) and pituitary stalk (39.7%) (Supplementary Table S5). A differential analysis of hemangioblastoma at each site showed that tumor size located in the sellar region and the optic nerve were generally smaller, which reached statistical significance compared with those located in the cerebrum (Supplementary Figure S1B). A correlation analysis was performed between tumor anatomical regions and symptoms. We found that patients with sellar lesions mainly experienced endocrine-related symptoms (Cramer's *V* = 0.33), and the optic nerve was strongly correlated with visual defect (Cramer's *V* = 0.53) (Supplementary Figure S1C).

Compared with solid tumors, cystic tumors are more prone to superficial location (*p* = 2.08 × 10^−3^) (Supplementary Table S6). A comparison of the incidence of edema by tumor sites revealed that edema was less common in sellar region lesions than in the cerebrum and optic nerve (*p* = 0.0003 and *p* = 0.012, respectively) (Supplementary Table S7). We also found a significant correlation between cysts and edema (*r* = 0.307, *p* < 0.05). More importantly, an analysis of tumor anatomical distribution of supratentorial cystic and solid tumors revealed that cystic tumors were mainly located in the cerebrum (62.5%) and the highest proportion of solid tumors was found in the sellar region (29.3%), and the proportion of cystic tumors in the cerebrum was significantly higher than that in the optic nerve (*p* = 4.78 × 10^−4^) and sellar region (*p* = 5.50 × 10^−5^) (Supplementary Table S8).

### 3.3. Cystic hemangioblastomas confer improved prognosis

**Figure 2:**
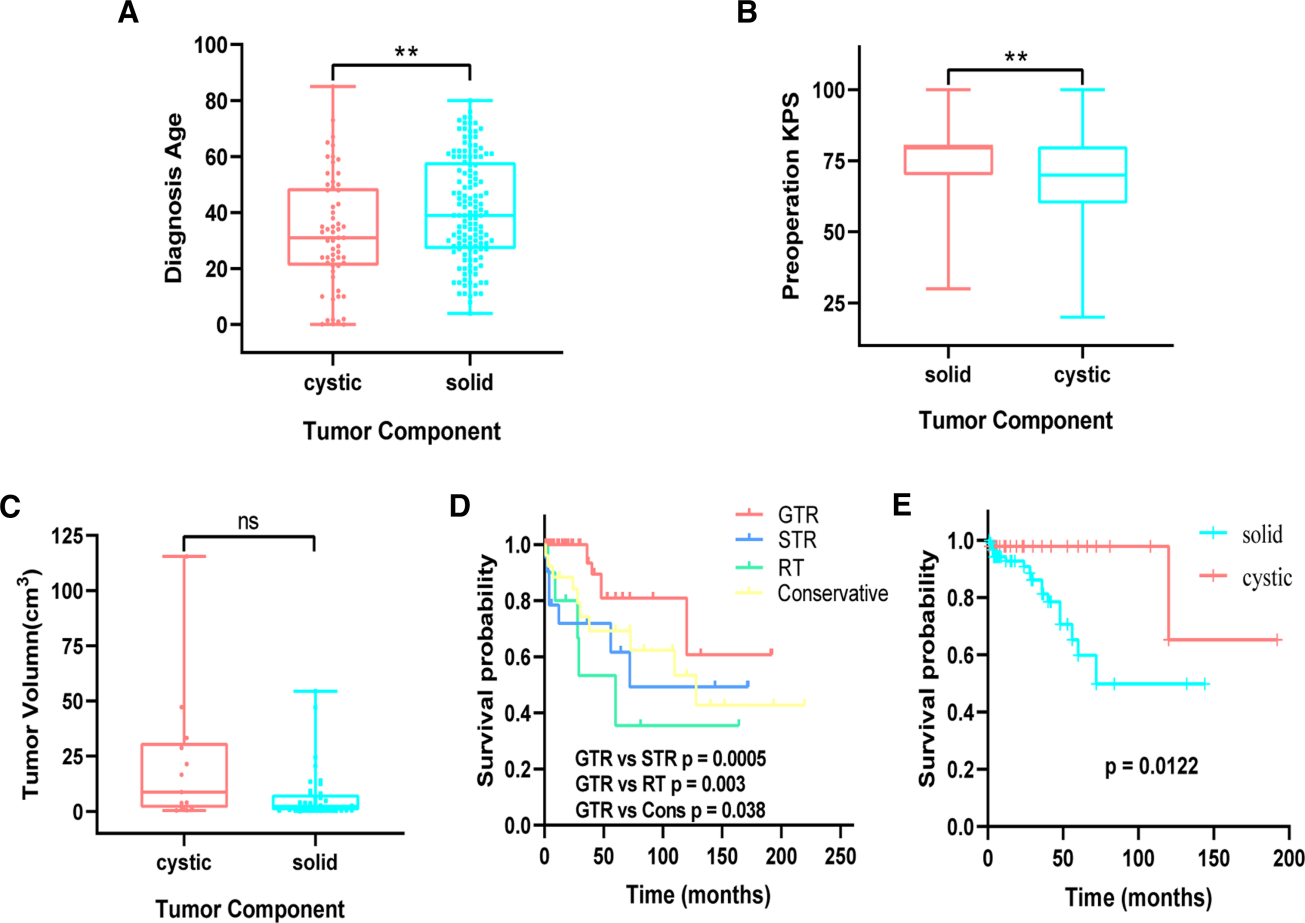
(**A**) comparison of age between cystic and solid patients. (**B**) Comparison of preoperation KPS between cystic and solid patients. (**C**) Comparison of cystic and solid tumor volume in purely supratentorial solitary hemangioblastoma. (**D**) Kaplan–Meier analysis of progression-free survival of supratentorial hemangioblastoma stratified by treatment modalities. GTR vs. STR (*p* = 0.0005), GTR vs. RT (*p* = 0.003). (**E**) Cystic vs. solid (*p* = 0.0122). KPS, Karnofsky Performance Scale; STR, subtotal resection.

Patients were divided into cystic and solid subgroups and then stratified by age subgroups; the mean age at initial diagnosis was found to be younger in cystic (33.35 ± 20.24 years) than in solid tumor (41.16 ± 18.58 years) (Figure 2A). A further analysis of each age subgroup revealed statistical significance between ≤10 years and other subgroups for cystic and solid tumors; that is, younger patients (≤10 years) were more likely to have cystic tumor (Supplementary Table S9). In addition, preoperative KPS (Figure 2B) and postoperative complications (Table 2) were analyzed in patients with cystic and solid tumors. We found that patients with cystic tumors had significantly lower preoperative KPSs than those with solid tumors (*p* = 0.001), but patients with cystic tumors had a significantly lower risk of developing complications than those with solid tumors (*p* = 0.012). There was an obvious trend that the volume of the cystic tumor was larger than that of solid tumor but with no significant difference (Figure 2C). Survival analysis was performed using the Kaplan–Meir survival curve, which was examined by employing the log-rank test. We found a clear benefit for GTR patients than for STR (log-rank, *p* = 0.0005) or radiotherapy (RT) patients with statistically significant values (log-rank, *p* = 0.003) (Figure 2D). Further grouped by cystic and solid status, Kaplan–Meir survival curves revealed that patients with cystic tumors had a significantly better prognosis than those with solid status (log-rank, *p* = 0.0122) (Figure 2E).

**Table 2:** Comparison of clinical features between cystic and solid cases.

Parameters	Tumor component		*χ*^2^/Z	*p*-value
	Cystic	Solid		
**Age at diagnosis (year)**				
Median (P25, P75)	32.00 (21.25, 49.75)	39.00 (27.00, 58.00)	2.473	0.013*
**Preop KPS score**				
Median (P25, P75)	70.00 (60.00, 80.00)	80.00 (70.00, 80.00)	3.474	0.001**
**Postop KPS score**				
Median (P25, P75)	80.00 (70.00, 80.00)	80.00 (70.00, 80.00)	0.646	0.518
**Sex, *n* (%)**				
Male	30 (47.6%)	65 (45.1%)	0.109	0.742
Female	33 (52.4%)	57 (54.9%)		
**Peritumoral edema**				
Yes	24 (54.5%)	31 (40.3%)	2.305	0.129
No	20 (45.5%)	46 (59.7%)		
**Infratentorial HB, *n* (%)**				
Yes	13 (25.0%)	46 (35.4%)	1.828	0.176
No	39 (75.0%)	84 (64.6%)		
**Postop complication**				
Yes	8 (25.0%)	38 (51.4%)	6.315	0.012*
No	24 (75.0%)	36 (48.6%)		
**Surgery**				
GTR	45 (86.5%)	84 (78.5%)	1.475	0.224
STR	7 (13.5%)	23 (21.5%)		
**Conservative**				
Yes	6 (10.2%)	13 (10.0%)	0.001	0.971
No	53 (89.9%)	117 (90.0%)		
**Radiotherapy**				
Yes	1 (1.7%)	10 (7.7%)	2.663	0.103
No	58 (98.3%)	120 (92.3%)		

STR, subtotal resection; HB, hemangioblastoma.

* *p* < 0.05; **p < 0.01.

### 3.4. Differences in clinical characteristics for supratentorial hemangioblastoma between sporadic and VHL patient groups

The patients were divided into two groups: VHL and sporadic. We compared the median onset age between both groups (Supplementary Table 10), which suggested that there was a significant difference between sporadic (median age: 42.50 years) and VHL-related hemangioblastoma (median age 33.00 years) (*p* = 0.010). A further analysis stratified by gender revealed that the median age of VHL patients (32.70 ± 12.82 years) was significantly lower than that of sporadic patients (42.15 ± 20.93 years) in the female category (*p* = 0.010) (Supplementary Table 10). In addition, those with supratentorial solitary lesion mainly belonged to the sporadic group (91.82%) (Supplementary Figure S2A). A comparison of tumor volumes between sporadic and VHL-related supratentorial hemangioblastomas revealed that the volume in the former was significantly larger than that of the latter (*p* < 0.0001) (Supplementary Figure S2B). Meanwhile, those with VHL differ from their counterparts in terms of symptomatic occurrence, ICP, infratentorial lesions, preoperative KPS, visceral lesion, retinal hemangioblastoma, and intratumoral hemorrhage (Supplementary Table 10). Furthermore, the difference in tumor location between the two groups was mainly reflected in the cerebrum and optic nerve (Supplementary Table 11). A correlation analysis revealed that VHL disease was negatively correlated with the cerebrum (Spearman *r* = −0.305, *p* < 0.05) and positively correlated with the optic nerve (Spearman *r* = 0.322, *p* < 0.01) but not with the sellar region (*p* = 0.439) and the ventricle (*p* = 0.713).

### 3.5. Risk factors for progression-free survival in supratentorial hemangioblastoma

We performed a univariate analysis for determining the potential risk factors associated with progression-free survival in patients with a supratentorial hemangioblastoma, such as gender, diagnosis age, symptom, VHL disease, infratentorial lesion, tumor component (solid and cystic), preoperation and postoperation KPS (<70 and ≥70), surgery strategy (GTR and non-GTR), and others. Of these factors, cystic tumor and GTR were significant predictors of longer PFS for supratentorial hemangioblastoma. Factors associated with longer control were symptoms (hazard ratio (HR): 4.26, 95% CI: 1.281–14.167, *p* = 0.018), cystic lesion (HR: 0.277, 95% CI: 0.095–0.803, *p* = 0.018), and GTR (HR: 0.243, 95% CI: 0.104–0.568, *p* = 0.001) in univariate analysis. These factors were then subjected to multivariate Cox regression analysis, and the result showed that cystic tumor (HR: 0.186, 95% CI: 0.043–0.803, *p* = 0.024) and GTR (HR: 0.126, 95% CI: 0.049–0.323, *p* < 0.001) was a significant independent predictor for improved PFS when compared with solid tumor and non-GTR (Table 3).

**Table 3:** Univariate and multivariate Cox regression analyses of potential risk factors influencing the PFS of patients with supratentorial hemangioblastoma.

Variate	Univariate analysis		Multivariate analysis	
	HR (95% CI)	*p*-value	HR (95% CI)	*p*-value
**Sex**				
Female	Reference level			
Male	1.851 (0.896–3.822)	0.096		
**Diagnosis age**				
≤20	Reference level			
20–50	1.038 (0.427–2.519)	0.935		
>50	1.118 (0.402–3.106)	0.831		
**Symptom**				
Yes	4.260 (1.281–14.167)	0.018*	3.968 (0.520–30.290)	0.184
No	Reference level			
**Preoperation KPS**				
<70	Reference level			
≥70	0.676 (0.229–1.992)	0.478		
**Postoperation KPS**				
<70	Reference level			
≥70	0.305 (0.090–1.037)	0.057		
**Solitary tumor in the supratentorial area**				
Solitary	0.448 (0.137–1.471)	0.186		
Multiple	Reference level			
**Retinal hemangioblastoma**				
Yes	1.418 (0.625–3.217)	0.403		
No	Reference level			
**Visceral lesions**				
Yes	0.907 (0.397–2.074)	0.817		
No	Reference level			
**VHL disease**				
No	Reference level			
Yes	0.833 (0.406–1.712)	0.620		
**Infratentorial lesion**				
No	Reference level			
Yes	1.254 (0.598–2.633)	0.549		
**Location**				
Deep	1.250 (0.654–2.387)	0.499		
Superficial	Reference level			
**Tumor component**				
Solid	Reference level			
Cystic	0.277 (0.095–0.803)	0.018*	0.186 (0.043–0.803)	0.024*
**Treatment**				
Non-GTR	Reference level			
GTR	0.243 (0.104–0.568)	0.001**	0.126 (0.049–0.323)	1.7 × 10^−5^**

PFS, progression-free survival; CI, confidence interval; KPS, Karnofsky Performance Scale; VHL, von Hippel–Lindau.

All factors with p < 0.05 in the univariate analysis were selected for multivariate Cox regression analysis.

* *p* < 0.05; **p < 0.01.

### 3.6. Supratentorial hemangioblastoma case cohort from Xiangya hospital

**Figure 3:**
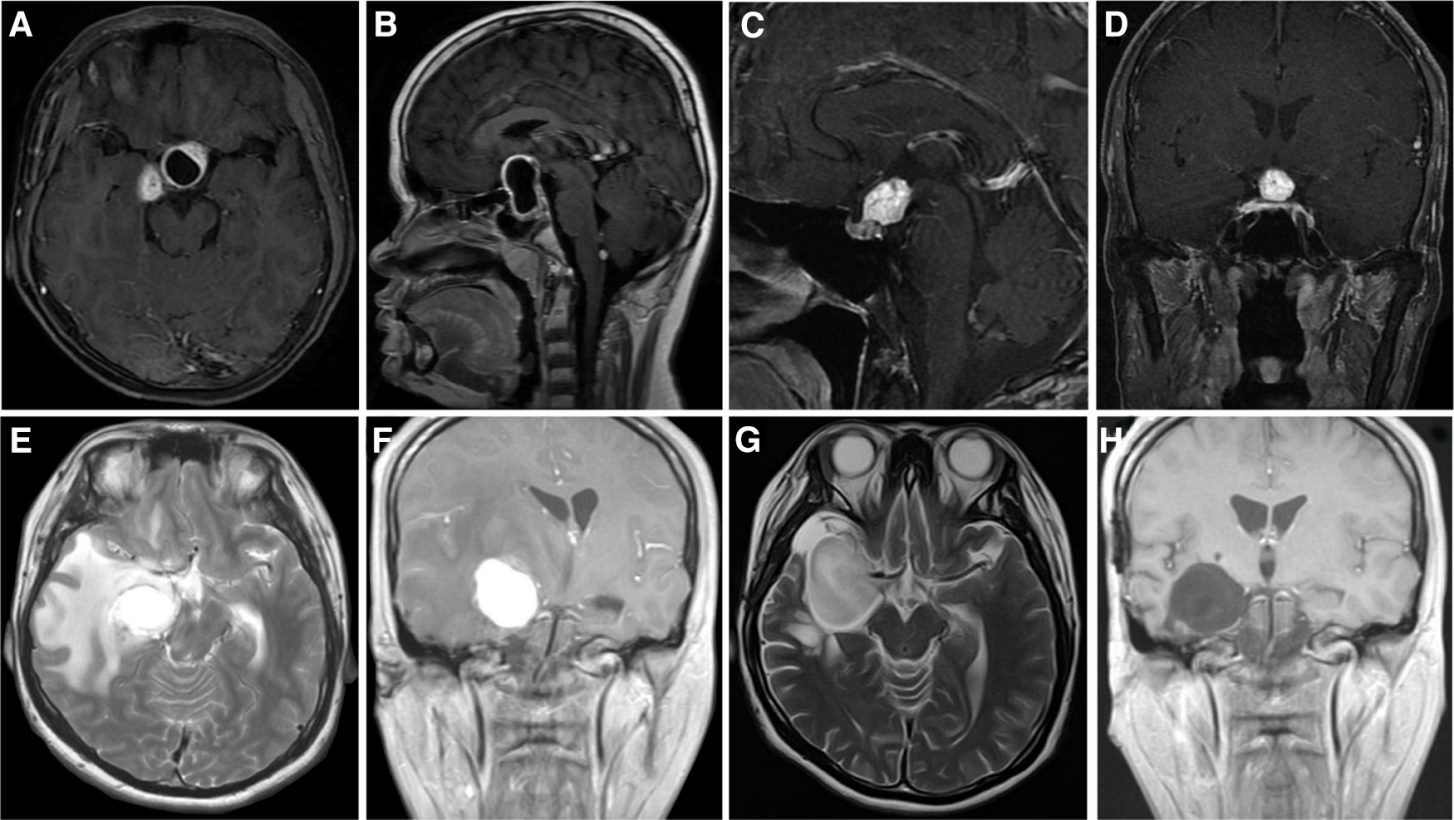
Imaging data of Xiangya cases. (**A,B**) Case 1: Cystic hemangioblastoma in the sellar region. (**C,D**) Case 3: Solid hemangioblastoma in pituitary stalk. Case 7: Solid hemangioblastoma in the basal ganglia region accompanied by massive edema (**E,F**), the edema subsided completely 3 weeks after operation (**G**), and there was no recurrence 26 months after operation (**H**).

We collected a cohort of 13 supratentorial hemangioblastoma patients diagnosed at the Xiangya Hospital, Central South University, between the years 2010 and 2021, with the breakup being four VHL-related and nine sporadic supratentorial hemangioblastomas. Patient clinical characteristics are given in Supplementary Table 1^2^, and representative patients with hemangioblastomas are described in Figure 3 and Supplementary Figure S3.

**Figure 4:**
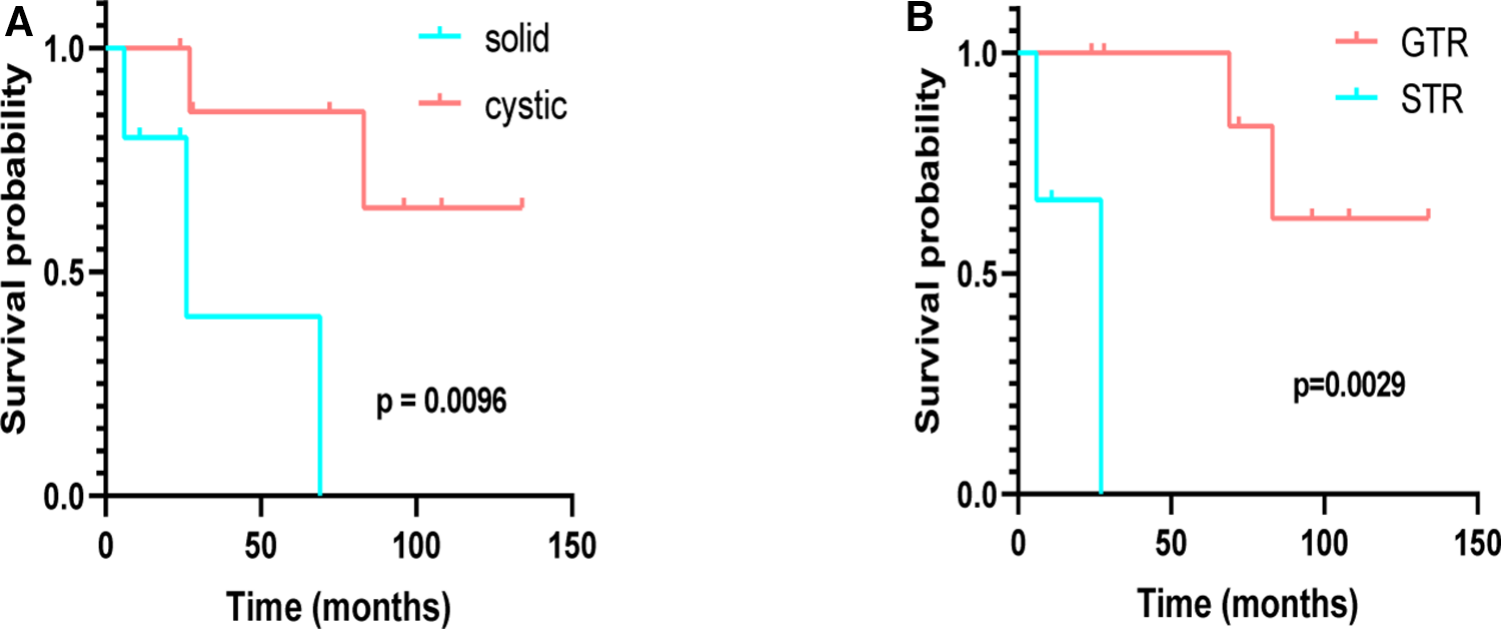
Kaplan–Meier analysis of progression-free survival of supratentorial hemangioblastoma stratified by treatment modalities. (**A**) Cystic vs. solid (*p* = 0.0096); (**B**) GTR vs. STR (*p* = 0.0029).

The median age at initial diagnosis was 36.38 ± 15.49 years in cystic and 37.40 ± 14.94 years in solid tumors, respectively (*p* = 0.909), and the mean preoperative KPS scores of cystic and solid tumors were 70.00 ± 9.26 and 82.00 ± 4.47, respectively (*p* = 0.022) (Supplementary Table 1^3^). Six patients were diagnosed with peritumoral edema around the tumor (five cerebrum and one hippocampal), and all give cerebrum patients who underwent surgical resection achieved GTR. Survival analysis was performed (K–M curves), which revealed that the PFS of patients with cystic supratentorial hemangioblastoma was significantly longer than that of patients with solid tumors, and the result was statistically significant (log-rank, *p* = 0.0096) (Figure 4A). Patients who underwent GTR had a better PFS than those who underwent STR, and the result was statistically significant (Figure 4B) (log-rank, *p* = 0.0029).

## 4. Discussion

CNS hemangioblastomas are the most common clinical manifestations of VHL disease. Previous studies have indicated that the growth rate of intracranial hemangioblastomas is substantially greater than that of other benign intracranial tumors (17, 18). However, CNS-associated hemangioblastomas are highly variable in supratentorial and infratentorial anatomical distribution. Lonser et al. (19) analyzed 225 VHL patients with CNS hemangioblastomas, and supratentorial patients accounted for 10.2% (23 cases in total). In terms of the number of tumors, there were 1921 tumors in 225 patients, of which 1% (21 supratentorial hemangioblastomas) were supratentorial locations. By analyzing 152 CNS-associated hemangioblastomas, Peyre et al. (7) showed that VHL accounted for 54% and supratentorial hemangioblastoma occurred in 3.2%, which is in agreement with reports on VHL patients, with supratentorial hemangioblastoma occurring in 1%–6% (14, 15). In addition, they found that only 13 (3.2%) of 409 VHL patients had supratentorial tumors, of which 4 were supratentorial multiple hemangioblastomas. A further analysis of the imaging data of 14 tumors revealed that 13 tumors had progressed (92.9%); in contrast, of 373 infratentorial cases reported by Wanebo et al. (20), only 44% reported tumor growth. Thus, based on the progression of supratentorial hemangioblastoma reported by Peyre et al. the possibility of supratentorial hemangioblastoma developing further seems to be higher (92.9%) than infratentorial and spinal hemangioblastoma (44%).

It is of great practical significance to clarify the clinical characteristics between cystic and solid components in hemangioblastoma. Our data showed that among supratentorial tumors, cystic tumors accounted for 27%, compared with the rate of 33% reported by Mills et al. (21). We further analyzed the location distribution of supratentorial cystic tumors and found that cystic tumors were mainly located in the cerebrum. Similar to cerebellar hemangioblastoma, which is mainly located in the posterior (74%) and superficial (68%) regions of the cerebellar cortex rather than in the anterior or deep region (6), supratentorial cystic hemangioblastomas are more frequently located in the superficial (58.2%) than in the deep cerebral cortex. This may be due to differences in the hydraulic conductivity of the cerebrum compared with other supratentorial locations. These intrinsic anatomic differences in fluid transport may make the cerebrum more prone to the generation of peritumoral cysts (22). In addition, peritumoral edema precedes and underlies the propagation of peritumoral and intratumoral cysts. Our data showed that there is a significant correlation between the presence of edema and the presence of cystic components in supratentorial tumors (*r* = 0.307, *p* < 0.01). Previous research has provided insights into the potential mechanisms leading to the formation and progression of tumor-related peritumoral cysts, which has an association with high intratumoral pressure, increased vascular permeability, and the levels of vascular endothelial growth factor (VEGF) (23, 24).

The formation of peritumoral edema or cyst is an important reason for the occurrence of symptoms of patients, but this edema will subside with tumor resection. For example, in patient 7 from our institute, the preoperative massive peritumoral edema disappeared after total tumor resection. Moreover, Peyre et al. (7) found no significant correlation between the formation of cysts and tumor size in a small series of cases. Similarly, consistent with their results, our data showed no correlation between supratentorial cyst formation and tumor size (*p* = 0.053). In view of its highly vascular characteristics, hemangioblastoma often shows obvious contrast enhancement on T1-MRI (25, 26). The signal of flow void within the tumor on the T2-weighted images is occasionally observed and regarded as a characteristic feature of the tumor (27, 28). These regions are considered to be intratumoral vascular structures. In our data, the hemangioblastoma with the flow void effect accounted for 13.5%. The typical vascular flow void signal was shown in patient 2, which is a low signal on T2 MRI and postcontrast enhancement. In addition, by analyzing the surgical resection degree of supratentorial tumors, we found that the total resection of cystic tumors located in the cerebral lobe could be easily performed, while for sellar tumors, whether cystic or solid type, the total resection of GTR was more difficult to perform compared with that of the other parts (29). Therefore, it is suggested that both cystic and solid tumors in the sellar region should be inspected before surgery to evaluate whether they can be completely resected.

Previous studies have confirmed that, during the assessment of the progression from the asymptomatic to the symptomatic state, the tumor size gradually increases, and the volume of the cyst is significantly larger than that of the tumor (20, 22). We found that in patients with supratentorial solitary tumor, the size of the cystic tumor was significantly larger than that of solid tumors; however, this result had no statistical difference. A further analysis of the difference in tumor size between symptomatic and asymptomatic hemangioblastoma showed that the mean size of symptomatic hemangioblastoma was larger than that of asymptomatic hemangioblastoma.

In our analysis, we combined STR and RT as an independent treatment modality to compare with GTR in extending PFS. The results showed that STR and RT were significantly inferior to GTR. In previous studies, high-dose fractioned external beam radiotherapy (EBRT) has been demonstrated to improve overall survival (OS) and disease-free survival (DFS) for CNS-related hemangioblastoma (30, 31). Recently, numerous studies of stereotactic radiosurgery (SRS) for CNS hemangioblastoma showed that local tumor control rates at 5 and 10 years ranged from 83% to 94% and 61% to 80%, respectively (32–34), with a 0%–7% risk for adverse radiation effects. Of these, Gamma Knife Radiosurgery (GKRS) is considered an acceptable treatment modality for CNS hemangioblastoma, the 5-year tumor control rate was 74%–85% (35, 36), and compared with patients with a solitary hemangioblastoma, those with multiple lesions in CNS were more likely to show progress after GKRS treatment (35). Liebenow et al. (37) held the idea that GKRS altered the clinical course from a saltatory growth pattern to a reduction of tumor size. They reported that the formation rates of new hemangioblastomas at 1, 3, and 5 years were 97%, 80%, and 46%, respectively. In addition, GKRS is not an ideal treatment for cystic hemangioblastoma (32, 34, 37), especially for small mural nodules with large cysts and for those who need urgent relief from symptoms (38). As shown in patient 11 from our institution (Supplementary Figure S3), changes occurring in tumors were highly uncertain during gamma knife treatment, and it was shown during dynamic observation that the tumor could shrink, stabilize, or increase in size, and even cystic changes occurred after this treatment. Gamma knife treatment did not prevent the development of new lesions. If the tumor appears to undergo a cystic change, surgery should be the only curative treatment modality.

However, it is worth noting that GKRS may be an effective treatment for cystic lesions, when they are located in an unfavorable surgical location, or for multiple intracranial lesions in an individual patient (39–41). Despite few studies having reported the efficacy of RT for supratentorial hemangioblastoma, studies on CNS hemangioblastoma have shown that the 5-year OS of the VHL subgroup was better than that of the non-VHL when patients were stratified by VHL status (32, 34). Evidence suggests that surgical resection can be the first line of treatment of supratentorial hemangioblastoma, and GTR should be the first priority. However, in those in whom lesions occur in the eloquent area or in whom only STR can be achieved, EBRT, SRS, or GKRS could be recommended as an alternative treatment modality for small, solid, or noncystic and VHL-associated lesions.

We explored the relationship between different age subgroups and cystic or solid tumors. The results showed that younger symptomatic patients tended to have cystic hemangioblastoma, while older symptomatic patients tended to have solid hemangioblastoma. Furthermore, survival analysis showed that the PFS of cystic patients was better than that of solid patients (*p* < 0.05). Combined with the Cox regression analysis, it was found that cystic tumor and GTR were two factors of PFS. Based on these results, we recommend that, for young patients with supratentorial cystic hemangioblastoma accompanied by edema, if the tumor is not located in the sellar region, for which the total resection rate was 65% in our study, which was lesser than that for cerebrum (89%), ventricle (69%), and optic nerve (80%), GTR could be the first priority for surgery for achieving a better prognosis.

Craniospinal hemangioblastoma occurs in over 80% of patients with VHL-associated hemangioblastoma, and multiple lesions occur in over 90% of patients (25). The results of our data analysis showed that sporadic patient cases (91.82%) were found for supratentorial single lesions. While sporadic and VHL-related hemangioblastomas share histological characteristics in the CNS, the clinical course of these tumors may differ significantly (42). Compared with sporadic patient cases, VHL patients, whose lesions were detected at a younger age, are more likely to develop multiple tumors. Lonser et al. (19) also found that in patients with VHL, new tumorigenesis seemed to correlate with younger age. Patients younger than 20 years were more likely to develop new tumors than those older than 40 years. In our study, we found that the average age of VHL patients developing supratentorial hemangioblastoma was younger than that of their sporadic counterparts, indicating that, during the lifetime of a VHL patient, the risk of new tumors decreases with age. This age-related tumorigenesis may be due to VHL gene missense mutated pVHL (VHL protein) (8), which still potently participates in the course of hypoxia inducible factor (HIF) degradation. However, due to the instability of the mutant protein, it is often degraded by chaperone proteins (e.g. Hsp70 and Hsp90) shortly after transcription (43). Previous studies have confirmed a progressive decline in the function of these chaperones with aging, which may contribute to the prolongation of missense VHL protein activity and attenuation of the new tumor growth (44). This age-related decline in proteasome function may partially account for the difference in the onset age of supratentorial hemangioblastoma between the VHL and the sporadic patient cases.

In conclusion, in this study, we mainly performed a detailed and in-depth analysis of the clinically relevant features of supratentorial hemangioblastoma by summarizing previous case reports from the literature and case series from our institute. We found that, in supratentorial hemangioblastoma, GTR significantly prolonged patients’ PFS compared with STR and RT. Meanwhile, we also found that cystic hemangioblastoma existed as a separate entity and had significant differences with solid tumors in terms of preoperative KPS, treatment modality, and PFS. However, due to the limited number of cases and insufficient clinical data in this study, further multicentric prospective studies and in-depth exploration are needed to refine the management of supratentorial hemangioblastoma.

## Data Availability

The raw data supporting the conclusions of this article will be made available by the authors, without undue reservation.
