# Supplementary figures and images for: Clinical characteristics, surgical management, and prognostic factors for supratentorial hemangioblastoma: A retrospective study
Source: Front Surg. 2023 Jan 24;9:1092140. doi: 10.3389/fsurg.2022.1092140 (PMC9902503; doi:10.3389/fsurg.2022.1092140)

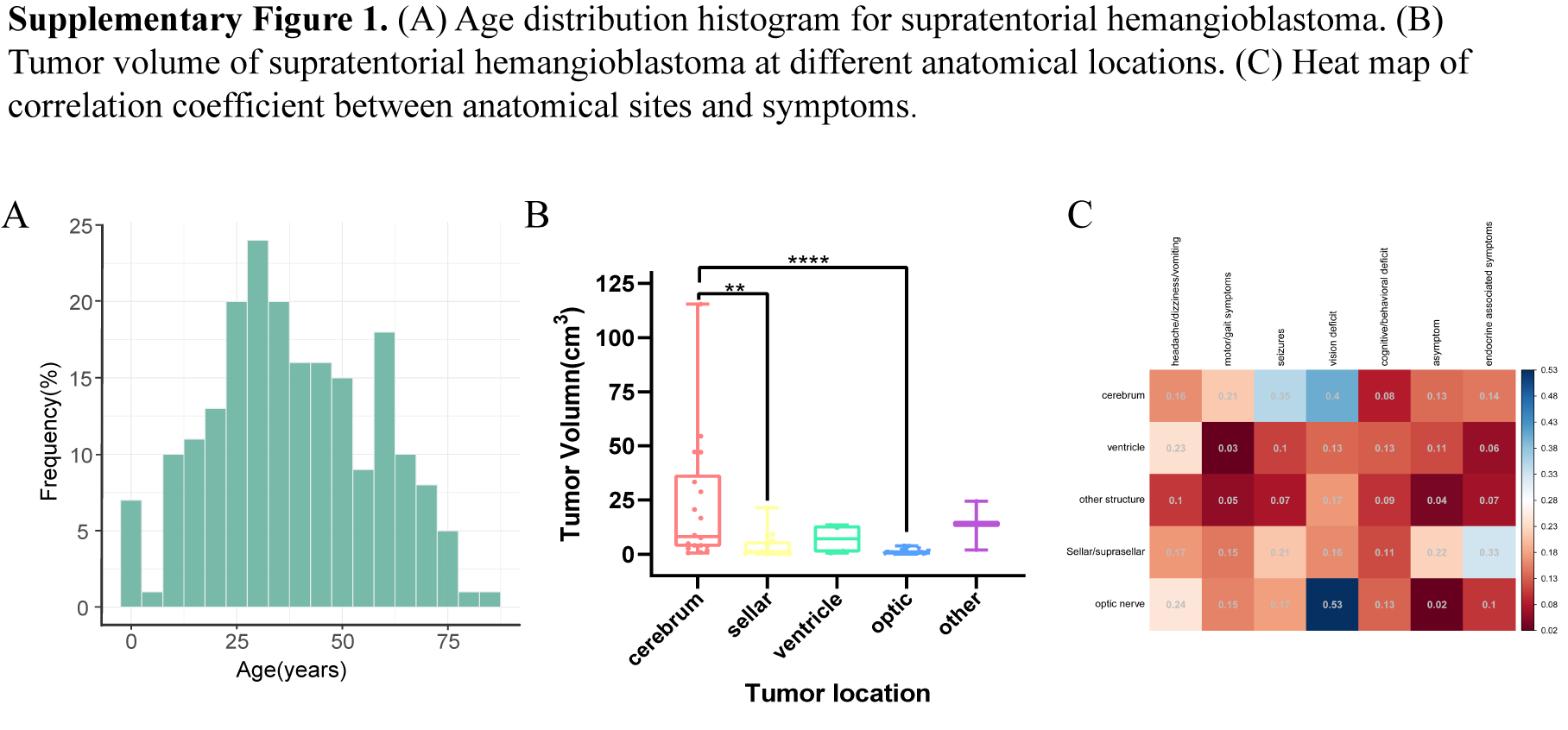

Supplement: Supplementary file 1 [file Image1.tif]

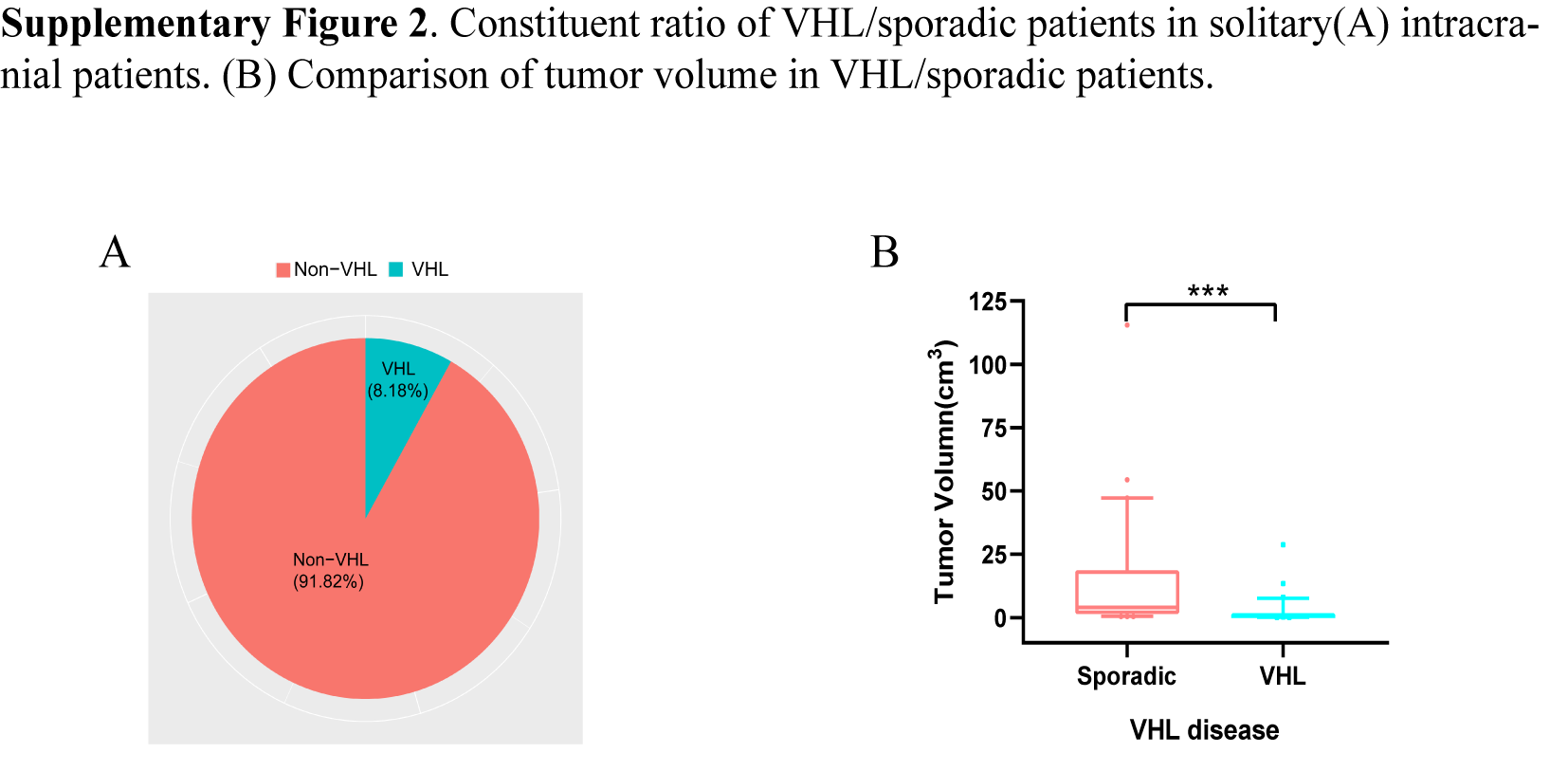

Supplement: Supplementary file 2 [file Image2.tif]

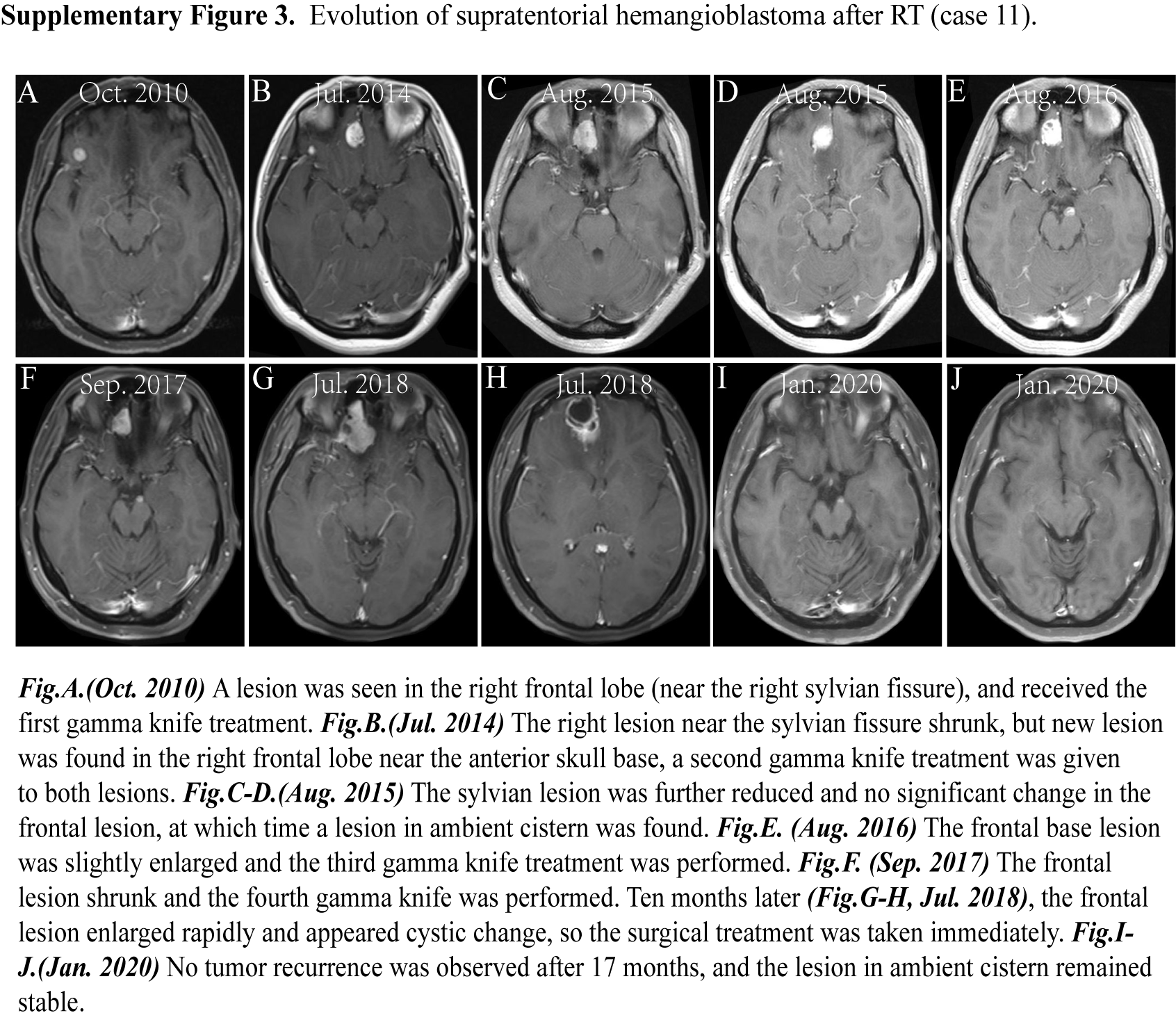

Supplement: Supplementary file 3 [file Image3.tif]
