# Supplementary material for: Clinical characteristics, surgical management, and prognostic factors for supratentorial hemangioblastoma: A retrospective study
Source: Front Surg. 2023 Jan 24;9:1092140. doi: 10.3389/fsurg.2022.1092140 (PMC9902503; doi:10.3389/fsurg.2022.1092140)
Supplement: Supplementary file 4 [file Table1.docx]

**Supplementary Table 1. Baseline characteristics of the study population.**

| **Parameters** | **Patients (n=237)**  **No. (%) [95% CI]** |
| --- | --- |
| Visceral lesions, No. (%)  Renal cell carcinoma  Adrenal phaeochromocytomas  Renal cyst  Pancreatic cyst  Pancreatic tumor  Liver cyst  Adrenal tumor  Paraganglioma  Renal tumor  Adrenal cyst  Endocrine pancreatic tumor  Pancreatic carcinoma  Spleen cyst  Spleen tumor  Prostate cyst  Ovarian cyst  Epididymal cyst  Cystadenoma of epididymis  Other diseases, No. (%)  None reported  Hypertension  Type II diabetes  Cerebral aneurysm  Gerstman's syndrome  Asperger’s syndrome  Chiari-Frommel syndrome  Type Ⅰ neurofibromatosis  Melanoma  Prostate cancer with lung metastasis  Breast cancer  Meningioma  Mental retardation  Ovary teratoma  Acoustic neuroma | 24/67  18/67  17/67  16/67  8/67  3/67  2/67  2/67  1/67  1/67  1/67  1/67  1/67  1/67  1/67  2/67  1/67  1/67  215/237 (91.0)  5/237 (2.1)  5/237 (2.1)  2/237 (0.8)  1/237 (0.4)  1/237 (0.4)  1/237 (0.4)  1/237 (0.4)  1/237 (0.4)  1/237 (0.4)  1/237 (0.4)  1/237 (0.4)  1/237 (0.4)  1/237 (0.4)  1/237 (0.4) |

**Supplementary Table 2. Imaging characteristics of the study population.**

| **Parameters** | **Patients (n=237)**  **No. (%) [95% CI]** |
| --- | --- |
| Main anatomical location  Cerebrum  Sella  Optic nerve  Ventricle  Others  CT-scan findings  Hypodense  Isodense  Hyperdense  Mixed  NA  Calcification  Yes  No  NA  Contrast enhancement (CT-scan and/or MRI)  Yes  No  NA  T1-weighted sequence MRI  Hypointense  Isointense  Hyperintense  Mixed  NA  T2-weighted sequence MRI  Hypointense  Isointense  Hyperintense  Mixed  NA  Dural attachment (CT-scan or MRI)  Yes  No  NA | 86 (36.3) [30.2-42.8]  58 (24.5) [19.1-30.5]  50 (21.1) [16.1-26.8]  19 (8.0) [4.9-12.2]  24 (10.1) [6.6-14.7]  9 (3.8) [1.4-6.2]  16 (6.8) [3.6-10.0]  20 (8.4) [4.8-11.9]  6 (2.5) [0.5-4.6]  186 (78.5)  6/237 (2.5) [1.9-14.8]  65/237 (27.4) [21.6-32.9]  166/237 (70.0)  173/237 (73.0) [66.9-78.5]  6/237 (2.5) [0.9-5.4]  58/237 (24.5)  13 (5.5) [2.6-8.3]  39 (16.5) [11.8-21.2]  5 (2.1) [0.2-4.0]  7 (3.0) [0.7-5.1]  173 (73.0)  1 (0.4) [0-1.3]  1 (0.4) [0-1.2]  57 (24.1) [19.1-29.3]  18 (7.6) [4.3-10.8]  160 (67.5)  25 (10.5) [6.6-14.5]  98 (41.4) [35.1-47.3]  114 (48.1) |

**Supplementary Table 3. Management characteristics of the study population.**

| **Parameters** | **Patients (n=237)**  **No. (%) [95% CI]** |
| --- | --- |
| Presence of clinical syndrome, No. (%)  Yes  No  NA  Symptom duration (month), No. (%)  < 6  6-12  13-60  > 60  NA  Preoperative misdiagnosis  Yes  No  NA  Embolization  Yes  No  NA  Tumor volume (mean) (cm^3^)  Postoperative clinical basic status in one week  Improvement  Stable  Deteriorate  Dead  Operation free  NA  Postoperative symptom changes in one week  Improvement  Stable  Deteriorate  Dead  Operation free  NA  Recurrence after GTR, No. (%)  Yes  No  NA  Progression free survival (PFS) (month), No. (%)  < 12  12-36  > 36  NA  Total follow-up (month), No. (%)  < 12  12-36  > 36  NA | 192 (81.0) [83.3-92.1]  27/237 (11.4) [7.2-15.5]  18 (7.6)  63 (26.6) [21.1-32.7]  30 (12.7) [8.7-17.6]  22 (9.3) [5.9-13.7]  8 (3.4) [1.5-6.5]  114 (48.1)  77 (32.5) [26.5-38.6]  24 (10.1) [6.4-13.9]  136 (57.4)  6 (2.5) [0.9-5.4]  211 (89.0) [84.3-92.7]  20 (8.4)  8.53 [4.1-13.0]  41 (17.3) [12.4-22.2]  70 (29.5) [23.9-35.3]  11 (4.6) [2.1-7.3]  9 (0.4) [0-1.3]  41 (17.3) [12.3-22.5]  73 (30.8)  52 (21.9) [16.9-27.3]  47 (19.8) [14.8-24.9]  19 (8.0) [4.6-11.5]  1 (0.4) [0-1.2]  38 (16.0) [11.7-20.7]  80 (33.8)  0/136 (0.0)  87/136 (64.0) [55.3-72.0]  48/136 (36.0)  53/237 (22.4) [17.2-28.2]  55/237 (23.2) [18.0-29.1]  61/237 (25.7) [20.3-31.8]  68/237 (28.7)  51/237 (21.5) [16.5-27.3]  51/237 (21.5) [16.5-27.3]  65/237 (27.4) [21.8-33.6]  70/237 (29.5) |

NA: Not available

**Supplementary Table 4. Distribution of lesion sites in different age subgroups**

| **Age subgroups (years)** | **Location** | **No. (%) [95% CI]** |
| --- | --- | --- |
| 0-20 | Cerebrum | 17 (0.472) [0.304-0.645] |
|  | Sellar | 8 (0.222) [0.101-0.392] |
|  | Ventricle | 6 (0.167) [0.064-0.328] |
|  | Optic nerve | 1 (0.028) [0.001-0.145] |
|  | Other structure | 4 (0.111) [0.031-0.261] |
| 20-50 | Cerebrum | 48 (0.432) [0.339-0.503] |
|  | Sellar | 19 (0.171) [0.106-0.254] |
|  | Ventricle | 8 (0.072) [0.032-0.137] |
|  | Optic nerve | 25 (0.225) [0.151-0.314] |
|  | Other structure | 11 (0.099) [0.051-0.170] |
| 50-85 | Cerebrum | 21 (0.362) [0.240-0.499] |
|  | Sellar | 17 (0.293) [0.181-0.427] |
|  | Ventricle | 4 (0.069) [0.019-0.167] |
|  | Optic nerve | 7 (0.121) [0.050-0.233] |
|  | Other structure | 9 (0.155) [0.073-0.274] |

**Supplementary Table 5. The specific anatomical location of supratentorial** **hemangioblastoma**

| **Location** | **No. (%)** |
| --- | --- |
| Cerebrum | n = 86 |
| Frontal | 28 (0.326) |
| Temporal | 27 (0.314) |
| Parietal | 18 (0.209) |
| Occipital | 7 (0.081) |
| Frontal + Temporal | 1 (0.012) |
| Temporal + Parietal + Occipital | 2 (0.023) |
| NA | 3 (0.035) |
| Sellar | n = 58 |
| Suprasellar | 26 (0.448) |
| Pituitary Stalk | 23 (0.397) |
| Intrasellar | 6 (0.103) |
| Paraselllar | 3 (0.051) |
| Ventricle | n = 19 |
| Lateral Ventricle | 13 (0.684) |
| Third Ventricle | 6 (0.316) |
| Optic Nerve | n = 50 |
| Other Location | n = 24 |
| Cerebral Falx | 4 (0.167) |
| Cistern | 1 (0.042) |
| Corpus Callosum | 4 (0.167) |
| Anterior Clinoid Process | 1 (0.042) |
| Trochlear Nerve | 1 (0.042) |
| Thalamus | 2 (0.083) |
| Pineal Region | 2 (0.083) |
| Basal Ganglia | 1 (0.042) |
| Anterior Commissure | 1 (0.042) |
| Trigeminal Nerve | 1 (0.042) |
| Anterior Skull Base | 1 (0.042) |
| Sphenoid Ridge | 1 (0.042) |
| Cerebral Aqueduct | 1 (0.042) |
| Multiple Lesion | 3 (0.125) |

**Supplementary Table 6. Comparison of clinical features between deep and superficial supratentorial hemangioblastoma**

| **Parameters** | **Tumor location** | | **χ^2^** | **p-value** |
| --- | --- | --- | --- | --- |
|  | **Superficial** | **Deep** |  |  |
| Tumor component |  |  |  |  |
| Cystic | 46 (39.0%) | 18 (19.4%) | 9.482 | 2.08E-03 |
| Solid | 72 (61.0%) | 75 (80.6%) |  |  |
| Peritumoral edema |  |  |  |  |
| Yes | 43 (63.2%) | 14 (25.5%) | 17.454 | 2.9E-05 |
| No | 25 (36.8%) | 41 (74.5%) |  |  |
| Symptom |  |  |  |  |
| Yes | 109 (90.8%) | 80 (83.8%) | 2.456 | 0.1117 |
| No | 11 (9.2%) | 16 (16.2%) |  |  |
| Increased ICP |  |  |  |  |
| Yes | 14 (11.6%) | 16 (16.2%) | 0.975 | 0.324 |
| No | 107 (88.4%) | 83 (83.8%) |  |  |
| Infratentorial lesion |  |  |  |  |
| Yes | 31 (29.0%) | 32 (39.5%) | 2.296 | 0.130 |
| No | 76 (71.0%) | 49 (60.5%) |  |  |
| Pre-op KPS |  |  |  |  |
| ≥ 70 | 68 (81.0%) | 70 (83.3%) | 0.162 | 0.687 |
| < 70 | 16 (19.0%) | 14 (16.7%) |  |  |
| Post-op complication |  |  |  |  |
| Yes | 16 (29.6%) | 32 (58.2%) | 9.013 | 0.003 |
| No | 38 (70.4%) | 23 (41.8%) |  |  |
| Visceral lesions |  |  |  |  |
| Yes | 41 (41.0%) | 26 (34.2%) | 0.844 | 0.358 |
| No | 59 (59.0%) | 50 (65.8%) |  |  |
| Flow void effect |  |  |  |  |
| Yes | 11 (28.9%) | 21 (47.7%) | 3.022 | 0.082 |
| No | 27 (71.1%) | 23 (52.3%) |  |  |
| Retinal HB |  |  |  |  |
| Yes | 23 (22.1%) | 17 (21.3%) | 0.020 | 0.888 |
| No | 81 (77.9%) | 63 (78.8%) |  |  |
| hemorrhage |  |  |  |  |
| Yes | 12 (12.9%) | 3 (3.9%) | 4.147 | 0.042 |
| No | 81 (87.5%) | 73 (96.1%) |  |  |
| Surgical modalities |  |  |  |  |
| GTR | 85 (85.9%) | 50 (72.5%) | 4.622 | 0.032 |
| STR | 14 (14.1%) | 19 (27.5%) |  |  |
| Conservative treatment |  |  |  |  |
| Yes | 17 (12.7%) | 14 (13.6%) | 0.042 | 0.838 |
| No | 117 (87.3%) | 89 (96.4%) |  |  |
| Radiotherapy |  |  |  |  |
| Yes | 6 (4.5%) | 7 (6.8%) | 0.604 | 0.437 |
| No | 128 (95.5%) | 96 (93.2%) |  |  |

**Supplementary Table 7. Comparison of peritumoral edema in each location**

| **Location** | **Edema** | | **χ^2^** | **p-value** |
| --- | --- | --- | --- | --- |
|  | **yes** | **no** |  |  |
| cerebrum | 27 (67.5%) | 17 (56.7%) | 0.862 | 0.353 |
| optic nerve | 13 (32.5%) | 13 (43.3%) |  |  |
| cerebrum | 27 (84.4%) | 17 (42.5%) | 13.117 | 0.0003 |
| Sellar | 5 (15.6%) | 23 (57.5%) |  |  |
| cerebrum | 27 (81.8%) | 17 (70.8%) | 0.952 | 0.329 |
| ventricle | 6 (18.2%) | 7 (29.2%) |  |  |
| optic nerve | 13 (72.2%) | 13 (36.1%) | 6.268 | 0.012 |
| Sellar | 5 (27.8%) | 23 (63.9%) |  |  |
| optic nerve | 13 (68.4%) | 13 (65.0%) | 0.051 | 0.821 |
| ventricle | 6 (31.6%) | 7 (35.0%) |  |  |
| sellar | 5 (45.5%) | 23 (76.7%) | 2.323 | 0.127 |
| ventricle | 6 (54.5%) | 7 (23.3%) |  |  |

The data segmentation method was used for pairwise comparison in SPSS，p < 0.005 was considered to be statistical significance.

**Supplementary Table 8. Comparison of cystic and solid tumors in different anatomical location**

| **Location** | **Tumor component** | | **χ^2^** | **p-value** |
| --- | --- | --- | --- | --- |
|  | **cyst** | **solid** |  |  |
| Cerebrum | 40 (85.1%) | 39 (54.2%) | 12.20 | 4.78E-04 |
| Optic Nerve | 7 (14.9%) | 33 (45.8%) |  |  |
| Cerebrum | 40(83.3%) | 39 (47.6%) | 16.25 | 5.50E-05 |
| Sellar | 8 (16.7) | 43 (52.4%) |  |  |
| Cerebrum | 40 (88.9%) | 39 (75.0%) | 3.079 | 0.079 |
| Ventricle | 5 (11.1%) | 13 (25.0%) |  |  |
| Optic Nerve | 7 (58.3%) | 33 (71.7%) | 0.296 | 0.587 |
| Ventricle | 5 (41.7%) | 13 (28.3%) |  |  |
| Sellar | 8 (61.5%) | 43 (76.8%) | 0.604 | 0.437 |
| Ventricle | 5 (38.5%) | 13 (23.2%) |  |  |

The data segmentation method was used for pairwise comparison in SPSS，p < 0.005 was considered to be statistical significance.

**Supplementary Table 9. Comparison of cystic and solid tumors in different age subgroups**

| **Tumor component** | **Age subgroups (years)** | | | | | **p-value** |
| --- | --- | --- | --- | --- | --- | --- |
|  | **0-10** | **10-20** | **20-40** | **40-60** | **60-85** |  |
| Cystic | 10_a_ (16.7%) | 3_b_ (5.0%) | 27_b_ (45.0%) | 15_b_ (25.0%) | 5_b_ (8.3%) | 7.81E-04 |
| Solid | 3_a_ (2.2%) | 18_b_ (13.3%) | 50_b_ (37.0%) | 37_b_ (27.4%) | 27_b_ (20.0%) |  |

Each subscript letter indicates a subset of age grouping categories. At the level of 0.05, there is no significant difference between the columns of these categories.

**Supplementary Table 10. Comparison of clinical features between VHL-related (yes) and sporadic (no) cases**

| **Parameters** | **VHL** | | **χ^2^ /t/ Z** | **p-value** |
| --- | --- | --- | --- | --- |
|  | **Yes** | **No** |  |  |
| Age at diagnosis(year) |  |  |  |  |
| Median (P25, P75) | 33.00 (24.25, 43.75) | 42.50 (26.75, 60.00) | 2.564 | 0.010 |
| Age stratified by sex |  |  |  |  |
| Male (Mean ± SD) | 37.47 ± 13.87 | 40.81 ± 22.36 | 0.765 | 0.447 |
| Female (Mean ± SD) | 32.70 ± 12.82 | 42.15 ± 20.93 | 2.617 | 0.010 |
| Tumor component |  |  |  |  |
| Cystic | 21 (24.4%) | 37 (33.0%) | 1.744 | 0.187 |
| Solid | 65 (75.6%) | 75 (67.0%) |  |  |
| Peritumoral edema |  |  |  |  |
| Yes | 22 (46.8%) | 30 (44.1%) | 0.081 | 0.776 |
| No | 25 (53.2%) | 38 (55.9%) |  |  |
| Symptom |  |  |  |  |
| Yes | 68 (72.3%) | 110 (99.1%) | 31.868 | 1.65E-08 |
| No | 26 (27.7%) | 1 (0.9%) |  |  |
| Increased ICP |  |  |  |  |
| Yes | 4 (4.3%) | 22 (19.6%) | 10.972 | 0.001 |
| No | 90 (95.7%) | 90 (80.4%) |  |  |
| Infratentorial lesion |  |  |  |  |
| Yes | 55 (84.6%) | 8 (7.2%) | 106.882 | 4.73E-25 |
| No | 10 (15.4%) | 103 (92.8%) |  |  |
| Pre-op KPS |  |  |  |  |
| ≥ 70 | 59 (95.2%) | 72 (76.6%) | 9.569 | 0.002 |
| < 70 | 3 (4.8%) | 22 (23.4%) |  |  |
| Post-op complication |  |  |  |  |
| Yes | 19 (55.9%) | 27 (38.6%) | 2.780 | 0.095 |
| No | 15 (44.1%) | 43 (61.4%) |  |  |
| Visceral lesions |  |  |  |  |
| Yes | 64 (83.1%) | 3 (3.1%) | 115.218 | 7.05E-27 |
| No | 13 (16.9%) | 93 (96.9%) |  |  |
| Flow void effect |  |  |  |  |
| Yes | 9 (36.0%) | 21 (40.4%) | 0.136 | 0.712 |
| No | 16 (64.0%) | 31 (59.6%) |  |  |
| Retinal HB |  |  |  |  |
| Yes | 39 (49.4%) | 0 (0.0%) | 63.652 | 1.49E-15 |
| No | 40 (50.6%) | 101 (100.0%) |  |  |
| Hemorrhage |  |  |  |  |
| Yes | 0 (0.0%) | 13 (13.8%) | 9.645 | 0.002 |
| No | 64 (100.0%) | 81 (86.2%) |  |  |
| Location |  |  |  |  |
| Superficial | 57 (53.3%) | 69 (60.0%) | 1.023 | 0.312 |
| Deep | 50 (46.7%) | 46 (40.0%) |  |  |
| Surgical modalities |  |  |  |  |
| GTR | 42 (82.4%) | 82 (78.8%) | 0.263 | 0.608 |
| STR | 9 (17.6%) | 22 (21.2%) |  |  |
| Conservative treatment |  |  |  |  |
| Yes | 29 (31.9%) | 2 (1.9%) | 33.835 | 6.00E-9 |
| No | 62 (68.1%) | 106 (98.1%) |  |  |
| Radiotherapy |  |  |  |  |
| Yes | 11 (12.1%) | 2 (1.9%) | 8.475 | 0.004 |
| No | 80 (87.9%) | 106 (98.1%) |  |  |

HB: hemangioblastoma.

**Supplementary Table 11. Comparison of tumor detailed anatomical location between VHL-related (yes) and sporadic (no) cases**

| **Parameters** | **VHL** | | **χ^2^** | **p-value** |
| --- | --- | --- | --- | --- |
|  | **yes** | **no** |  |  |
| Cerebrum |  |  |  |  |
| Yes | 22 (20.6%) | 57 (49.6%) | 20.342 | 6.00E-06 |
| No | 85 (79.4%) | 58 (50.4%) |  |  |
| Ventricle |  |  |  |  |
| Yes | 7 (6.5%) | 10 (8.7%) | 0.364 | 0.547 |
| No | 100 (93.5%) | 105 (91.3%) |  |  |
| Sellar |  |  |  |  |
| Yes | 31 (29.0%) | 27 (23.5%) | 0.867 | 0.352 |
| No | 76 (71.0%) | 88 (76.5%) |  |  |
| Optic nerve |  |  |  |  |
| Yes | 39 (36.4%) | 11 (9.6%) | 22.956 | 2.00E-06 |
| No | 68 (63.6%) | 104 (90.4%) |  |  |
| Other structure |  |  |  |  |
| Yes | 9 (8.4%) | 10 (8.7%) | 0.006 | 0.940 |
| No | 98 (91.6%) | 105 (91.3%) |  |  |

**Supplementary Table 12. Supratentorial hemangioblastoma case cohort from Xiangya hospital**

| **Case** | **Sex** | **Age at diagnosis (years)** | **VHL** | **Localization** | **Tumor component** | **Edema** | **Treatment** | **Follow-up (month)** | **PFS status** | **Final outcome ^a^** |
| --- | --- | --- | --- | --- | --- | --- | --- | --- | --- | --- |
| Case 1 | Male | 15 | Yes | Sella | Cystic | No | STR | 27 | Progression | AWD |
| Case 2 | Male | 30 | No | Sella | Cystic | No | GTR | 72 | Stable | ADF |
| Case 3 | Male | 55 | No | Sella | Solid | No | STR | 6 | Progression | ADF |
| Case 4 | Male | 29 | Yes | Sella | Solid | No | STR | 11 | Stable | AWD |
| Case 5 | Male | 23 | No | Cerebrum (right) | Cystic | Yes | GTR | 28 | Stable | ADF |
| Case 6 | Male | 49 | Yes | Cerebrum (left) | Solid | No | GTR | 69 | Progression | AWD |
| Case 7 | Female | 18 | No | Basal ganglia (right) | Solid | Yes | GTR | 24 | Stable | ADF |
| Case 8 | Male | 60 | No | Cerebrum (right) | Cystic | No | GTR | 24 | Stable | ADF |
| Case 9 | Male | 55 | No | Cerebrum (left) | Cystic | Yes | GTR | 83 | Progression | ADF |
| Case 10 | Male | 43 | No | Cerebrum (left) | Cystic | Yes | GTR | 108 | Stable | ADF |
| Case 11 | Female | 36 | Yes | Cerebrum (right) | Solid | Yes | RT | 26 | Progression | AWD |
| Case 12 | Male | 36 | No | Cerebrum (left) | Cystic | No | GTR | 96 | Stable | ADF |
| Case 13 | Female | 29 | No | Cerebrum (left) | Cystic | Yes | GTR | 134 | Stable | ADF |

^a^ **ADF**, alive without disease; **AWD**, alive with von Hippel-Lindau disease.

**Supplementary Table 13. Supratentorial hemangioblastoma cases from Xiangya hospital cohort**

| **Variates** | **Tumor component** | | **χ^2^ / t** | **p-value** |
| --- | --- | --- | --- | --- |
|  | **Cystic (n=8)** | **Solid (n=5)** |  |  |
| Age at diagnosis (year) |  |  |  |  |
| Mean ± SD | 36.38 ± 15.49 | 37.40 ± 14.94 | 0.118 | 0.909 |
| Sex, n (%) |  |  |  |  |
| Female | 1 (12.5) | 2 (40.0) | 0.219 | 0.640 |
| Male | 7 (87.5) | 3 (60.0) |  |  |
| Pre-op KPS, n (%) |  |  |  |  |
| Mean ± SD | 70.00 ± 9.258 | 82.00 ± 4.472 | 2.677 | 0.022 |
| Post-op KPS, n (%) |  |  |  |  |
| Mean ± SD | 77.50 ± 4.629 | 76.00 ± 5.477 | 0.531 | 0.606 |
| VHL syndrome, n (%) |  |  |  |  |
| Yes | 1 (12.5) | 3 (60.0) |  | 0.217 |
| No | 7 (87.5) | 2 (40.0) |  |  |
| Infratentorial HB, n (%) |  |  |  |  |
| Yes | 1 (12.5) | 1 (20.0) |  | 0.641 |
| No | 7 (87.5) | 4 (80.0) |  |  |
| Treatment | 8 | 4 |  |  |
| GTR | 7 (87.5) | 2 (50.0) |  | 0.236 |
| STR | 1 (12.5) | 2 (50.0) |  |  |

HB: hemangioblastoma.
